# Lipid extraction and analysis of microalgae strain *pectinodesmus* PHM3 for biodiesel production

**DOI:** 10.1186/s12896-023-00784-8

**Published:** 2023-07-10

**Authors:** Dania Akram Kiyani, Shafia Maryam, Sundus Jabeen Amina, Abdullah Ahmad, Muhammad Waqas Alam Chattha, Hussnain Ahmed Janjua

**Affiliations:** 1 Department of Industrial Biotechnology, Atta–ur-Rahman School of Applied Biosciences, National University of Sciences and Technology (NUST), Islamabad, 44000 Pakistan; 2 Department of Plant Biotechnology, Atta–ur-Rahman School of Applied Biosciences, National University of Sciences and Technology (NUST), Islamabad, 44000 Pakistan

**Keywords:** Algae, Lipids, Fourier transmission infrared spectroscopy, Gas chromatography, Mass spectrometry, Biodiesel

## Abstract

The current study is focused on the lipid extract of microalgae; *Pectinodesmus* strain PHM3 and its general analysis in terms of chemical contents. Combinations of both chemical and mechanistic approaches were applied to obtain the maximum yield of lipids which was recorded to be 23% per gram through continuous agitation using Folch solution. The extraction methods used in this study included: Bligh and Dyers method, Continuous agitation method, Extraction using Soxhlet and Acid base extraction method. Lipid quantification of ethanol and Folch solution lipid extract was performed through gravimetric methods and qualification was done through Fourier Transmission Infrared Spectroscopy (FTIR) and Gas Chromatographymass spectrometry (GC-MS). Phytochemical analysis identified other compounds in ethanol extract and the results confirmed the presence of steroids, coumarins, tannins, phenols and carbohydrates. Transesterification of lipids showed 7% per gram dry weight yield of *Pectinodesmus* PHM3. GC-MS studies of extracted biodiesel suggested that 72% of biofuels was in the form of dipropyl ether, ethyl butyl ethers, methyl butyl ether and propyl butyl ether. Lipid processing of acid-base extract showed that oily nature of lipid shifted to a more precipitated form which is a common observation when mixture of lipids is converted to phosphatides.

## Introduction

With the expanding demand for alternative fuels, certain substitutes for fossil fuel have emerged recently and several are in their early stages of development [1]. One of the most significant alternatives to depleting fossil fuels is biodiesel; the use of which has several inherited advantages [2]. Being similar to conventional fuel with respect to its physical and chemical characteristics, diesel engines need no modifications for using biodiesel as a fuel [3]. Biodiesel is extremely eco-friendly non-hazardous and sustainable [4]. The combustion of biodiesel produces insignificant amount of waste gases in comparison to those of conventional fuel [5]. Nevertheless, the complications like augmented NOx release [6] and reduced oxidative stability [7] is a major concern for scientists. The biofuel produced from plant sources like grape seeds [8] and sunflower seeds [9] is referred to as the first generation biofuel. The problem with the first-generation fuels is that there is not enough arable land available to meet the fuel demands [10]. Production of these first-generation biofuels also cause a rise in the cost of animal feed and consequently that of human food [11]. The second generation of biofuel is produced from cellulose extract from sawdust, agricultural debris (corn stalks, fast-growing grasses, woody materials) and construction debris etc. This type of biofuel is advantageous as it does not cause any interference in food production and these crops can be grown on unfertile land as well [12]. The third generation of biofuels includes fuels formed from algal and cyanobacterial biomass [13].

Microalgae have been known to produce more oil content as compared to oil crops [14]. The oil yields of microalgae have been reported as high as 136,900 L/ha as compared to 5950 L/ha of Oil palm [15]. This percentage of fatty acids is higher than most plants’ lipid or oil content and makes algae favorable for biodiesel production. Other advantages of using microalgae for biofuel production include great photosynthetic productivity and increased production of biomass [16]. Microalgae do not require special environmental conditions and can grow without competing for land and even in extreme conditions. Advanced research technologies are focused on enhancing microalgae’s lipid content [17] and various techniques have been developed for algal culturing [18, 19]. During the prime growth conditions microalgae yield membrane lipids which include glycosyl glycerides and phosphoglycerides. Polyunsaturated fatty acids like palmitic acid and oleic acid undergo aerobic desaturation and chain elongation to form glycerolipids in the membrane [20]. Biofuel from algal biomass can be used in the motor car industry with little to no modifications or mixed with petroleum-based fuel.

Numerous mechanical methods are used for extracting lipids from microalgae include ultrasonic extraction, high-pressure homogenizer and hydrothermal liquefaction. On the contrary, three major types of chemical methods are used for the same purpose. One method involves two stages in which oil is first extracted using organic solvent that is subsquently converted to biodiesel with the help of a catalyst, like an acid, [21, 22] a base, [23, 24] or an enzyme [25]. The other method involves the use of acid to produce biodiesel from algal biomass which is carried out at atmospheric pressure and room temperature [26–28]. The third and last approach involves one-step conversion of biomass to biodiesel in the presence of high pressure and high temperature without using any catalyst [29, 30]. Each, method comes with its own advantages and disadvantages. To determine the potential of microalgae biomass as an alternative for biodiesel production, rigorous attempts are needed for detailed characterization of algae biomass, algae oil and algae-based biodiesel as very little information is available in the literature on the same. Multiple methods consisting of different techniques such as flocculation, floatation, membrane filtration, electrolysis and ultrasound are reported in the literature for lipids/biodiesel extraction from microalgae [31, 32].

The current study dealt with five different species of algae: *Dictyosphaerium* sp. (DHM1), *Dictyosphaerium* sp. (DHM2), *Pectinodesmus* sp. (PHM3), *Dictyosphaerium* sp. (DHS) and *Dictyosphaerium* sp. (DHSYM), with the aim of evaluating their potential for lipids and biodiesel production. *Dictyosphaerium* sp. The species of microalgae have not been explored previously for biofuel production. *Dictyosphaerium* sp. have been proved to capture and reduce carbon dioxide (CO_2_) [86]. The lipid content of all five microalgae specimens defines the future directive to use them for biofuel production. The lipid content in microalgae is influenced by growth conditions, extraction techniques and processing of extracts. Change in cultivation modes increases biomass and lipid contents [83]. Combination of various solvent extractions methods along with the analytical techniques such as GC-MS were applied to screen the algae with maximum lipid contents, PHM3 species was further selected for further investigations which may represents first report related to PHM3 for lipid extraction and subsequent biodiesel synthesis.

## Materials and methods

Autoclaved distilled water was used to make dilutions of already sterilized stock chemicals. For solutions, 10% ammonium solution, 2 N sodium hydroxide (NaOH) solution, 5% ferric chloride solution (FeCl_3_), ethanol, H_2_SO_4_ solution, hexane and methanol were purchased from Sigma Aldrich. Chloroform, 10% ferric chloride solution and 10% NaOH solution were purchased from Fischer Scientific. Deionized water, distilled water was purchased locally. Folch solution used for lipid extraction was prepared using chemical grade chloroform and methanol which were mixed in 2:1 (v/v) ratio in a sterile bottle. The bottle was sealed and kept in the dark, at room temperature.

### Biomass harvest

**Fig. 1:**
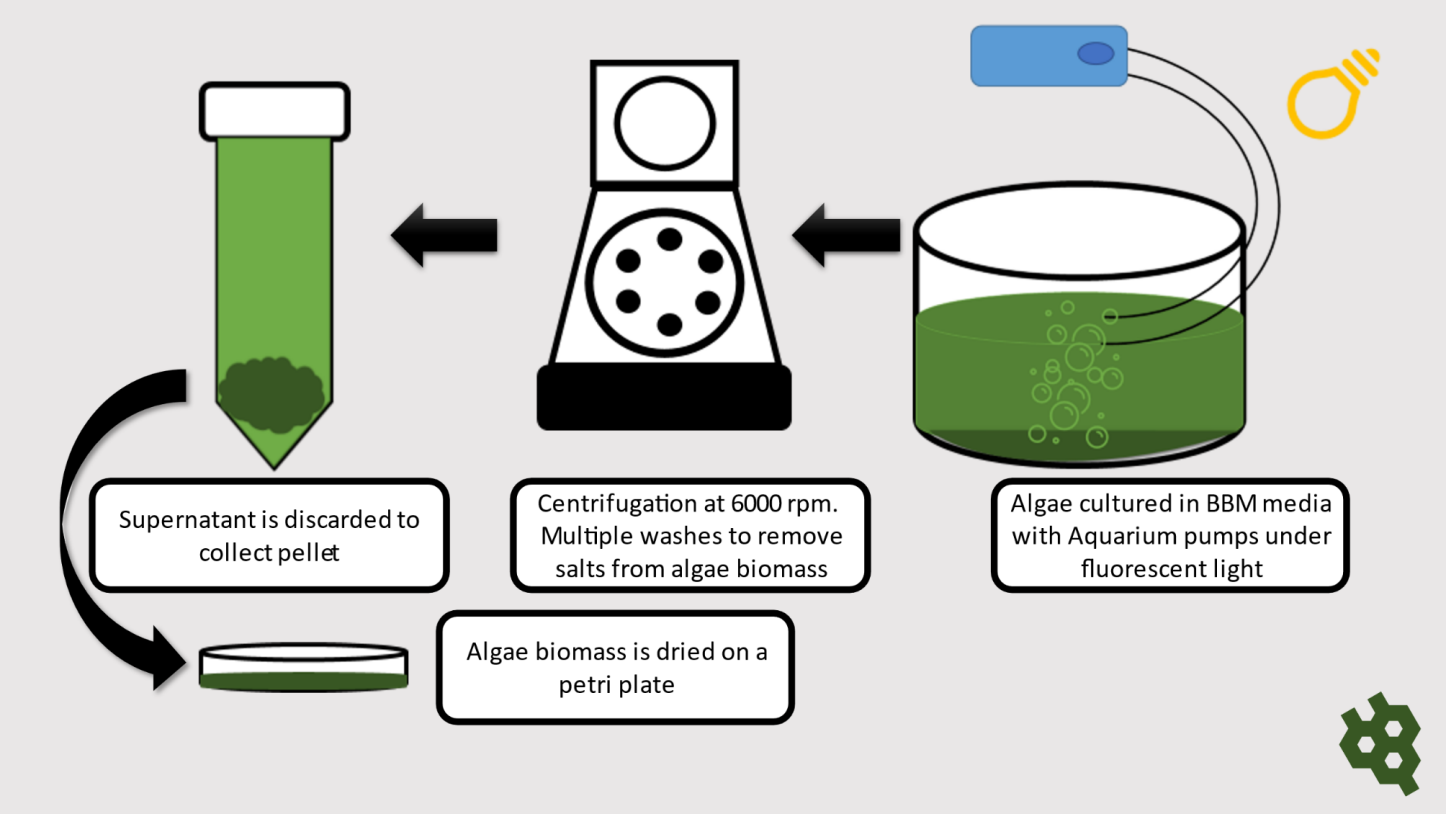
Schematic presentation of Algae Biomass harvestation

*Dictyosphaerium* sp. DHM1, *Dictyosphaerium* sp. DHM2, *Pectinodesmus* sp. PHM3 previously collected, identified and reported by Khalid et al. [33] and two other strains, *Dictyosphaerium* sp. DHS collected from the samples of the Institute of Environmental Engineering and Science, NUST, Islamabad and *Dictyosphaerium* sp. DHSYM isolated from the waste water of Fauji Fertilizers company were sub-cultured in liquid bold basal media (BBM) using an earlier described procedure [34]. Flask containing 20 ml algal inoculum, 920 ml autoclaved distilled water and 50 ml BBM were exposed to 36 W TLD white florescent lamp at 10µmol photons m^− 2^s^− 1^ with 24 h continuous light cycle. Aquarium air pumps were used for constant aeration at 26–28˚C. The cultures were allowed to grow for 1 month, with pH maintained at 7. The culture tanks were removed from their setups and algae-water mixture was collected into centrifuge tubes and centrifuged at 6000 rpm to settle down the biomass. The supernatant was decanted and pellet was again shaken and centrifuged with purified water to remove extra salts. After washing, the pellet was shifted into a glass dish and dried overnight in an incubator at 70°C to remove all moisture. This has been schematically presented in Fig. 1. The dried biomass was scraped, weighed, placed in a sealed container and stored in a refrigerator for further analysis of lipids contents and ultimately conversion into biodiesel.

### Lipid extraction

Prior to extraction of lipids, algal biomass was sonicated for 10 min at high intensity using a probe sonicator. Ultra-sonication increases vibrations in the cell walls which helps destroy the cell structure. The cells were lysed and lipids were more easily dispersed in the solvent.

#### Bligh and Dyers method for comparison of lipid yield from microalga strains

Modified version of previously described method [35] was used for lipid extraction in which a mixture of 5:4:10 of chloroform, distilled water and methanol respectively were added to a cylinder containing 1 g of dried algal biomass. Folch, Bligh and Dyer method with chloroform and methanol is a gold standard bench scale analysis for lipid analysis [87]. The samples were placed in shaking incubator at 37ºC overnight. This allowed the solution to thoroughly dissolve lipids. Subsequently, equal amount of Folch solution and water were added to the blend.

The cylinders were then centrifuged at 6000 rpm for 10–15 min. Three layers were created, the upper layer of methanol and water with polar lipids, the middle layer with cell debris and the final layer with chloroform carrying the major nonpolar lipid content. Supernatant was saved for FTIR analysis. The debris were discarded and the lipid layer was dried, weighed, dissolved in chloroform and kept at 4ºC for further analysis and experimentation, the experiment was run in triplicates for precise results.

#### Continuous agitation method

A modified method of ethanol extraction put forward by Han et al., [36] and Fajardo et al.,[37] was used in which 5 g of algae mass was mixed with 70ml of ethanol (95% v/v) in a container kept at 60–70ºC on a magnetic stirrer for 8 h approximately. The method insured increased surface contact of algae biomass to solvent, which resulted in increased yield. The extract was allowed to cool and centrifuged at 6000 rpm for 15 min. The extract was subsequently passed through a membrane filter to remove impurities. The lipid layer was dried, weighed, dissolved in chloroform and kept at 4ºC for further analysis and experimentation. Experiment was performed in triplicates for precise results. Same experiment was repeated with Folch solution as the solvent.

#### Extraction using soxhlet apparatus

Of all conventional strategies soxhlet extraction yields the highest amount of oil [88]. A modified protocol from previous study [38] was used in which ten parts algae was crushed and put in the thimble of the soxhlet apparatus. With 100ml of ethanol added in the apparatus, reflux was run at 70ºC for 12 h. Ethanol was added according to the required volume. After 12 h the solvent separated and dried. The lipid layer was weighed and stored. The same experiment was run with Folch solution as the solvent.

#### Acid base extraction

Modified version of acid base extraction initially developed by Duongbia et al. [39] and Sathish et al. [40] was used for chemical extraction of lipid from algal cells. In the first step, acid base hydrolysis was carried out in which 2ml of 1 M H_2_SO_4_ solution was added to 200 mg of algal dry mass in a falcon tube. The tubes were put in a shaking water bath at a temperature of 90ºC for 30 min and mixing was performed by shaking tubes every 15 min for uniform distribution of acid solution. Subsequently 2ml of 5 M NaOH solution was added to the tubes which were shaken and heated at 90˚C for 30 min. The tubes were shaken every 15 min for proper dispersal of the base solution. The samples were cooled and centrifuged to pellet the residual algal biomass [39].

The resulting supernatant phases were removed, collected from each sample in separate tubes and residual hydrolyzed biomass pellets were vigorously mixed with 2ml of deionized water. Resulting suspension was re-centrifuged, liquid phase was removed and again added to the corresponding tubes containing original supernatant. The second step involves chlorophyll precipitation. To the supernatant phases collected in the previous step, 3ml of 0.5 M H_2_SO_4_ was added which created a solid green precipitate as when the pH goes under 7, fatty acids revert to free form and create a white precipitate the lipids subsequently form a complex with the precipitated solids. The mixture was centrifuged, supernatant was removed and precipitates were collected. Five parts chemical grade hexane was added to the precipitate in the tubes. The tubes were then heated in water bath at 90ºC thus allowing the lipids to dissolve in the hexane [40].

The hot samples from the last step were chilled at 4^o^C and centrifuged subsequently. The hexane portion of the sample was removed and shifted to the clean flasks. The solid phase was removed from the system as a pellet of solid debris. Extract was passed through a membrane filter to remove solid impurities and the lipid layer was dried, weighed, dissolved in hexane again and kept at 4ºC for further analysis, experiments were performed in triplicates.

### Quantification of lipid yield

Percentage lipid yield was calculated using general formula for the quantification analysis [41]. The mass of lipids obtained was measured through a weighing balance.

### FTIR of lipid

Presence of various functional groups in multiple lipid extracts from various methodologies was confirmed using FTIR (IRAffinity-1 S). Small amount of lipids were mixed with KBr, a disc of the mixture was created by adding pressure. Infrared rays were passed through the disc and an FTIR spectrum of their absorbance was generated. Wavelength for scanning was set in the region of 500-4000 cm^− 1^.

### Phytochemical analysis of ethanol extract

Phytochemical testing of different extracts was checked through standardized method [84, 85]. This test confirms the presence or absence of terpenoids, alkaloids, coumarins, phenols, flavonoids, saponins, tannins, steroids, glycosides and carbohydrates.

#### Steroid test

The Liebermann-Burchard test was used to detect steroids [42]. To a 1:2 mixture of sulphuric acid and chloroform respectively, 0.5ml of algae extract was added. Positive result is confirmed through the formation of a red brown ring at the top of the algal extract.

#### Tannin test

Ferric chloride or tannin test was performed to indicate the presence of phenolic compounds [43]. This test uses 1ml 5% of FeCl_3_ added to 1ml of algal extract. Positive result for the presence of tannins is indicated through the creation of dark green, black, or blue color.

#### Saponin test

For the Saponin test, 2ml of water and 3ml of algae extract were mixed together in a graduated cylinder and shaken for 15 minutes [44]. Positive result is indicated through the formation of a foamy layer at around 1cm above the mixture.

#### Flavonoid test

Flavonoids are antioxidants normally found in plants. To perform the test, 2ml of algae extract and 1ml of sodium hydroxide were mixed together. In the presence of flavonoids, the mixture color is changed to yellow [45].

#### Phenol test

In this test, 1ml of algal extract, 2ml distilled water and 10% percent ferric chloride were mixed together. The ferric chloride was added drop wise. Positive result is indicated by the appearance of blue or green color in the algal extract [43].

#### Coumarin test

Coumarins are a family of benzopyrones present in plants and algae these are highly effective against viral disease, cancers, diabetes and cardiovascular diseases. In this test 1ml of algae extract and 1ml of 10% NaOH were mixed together. Appearance of yellow color confirms the presence of coumarins [46].

#### Test for glycosides

To test the presence of glycosides, 3ml chloroform was mixed with 10% ammonium solution and 2ml algal extract. Positive result is indicated with the development of pink color in the algal extract [47].

### Gas chromatography analysis of ethanol extract

The dried algal biomass (100 mg) of *Pectinodesmus* sp. (PHM3) was extracted using ethanol and water in 50:50 ratio. The impure organic part obtained from extraction was sieved using 0.22 μm membrane filter and concentrated to dryness using rotary evaporator machine at low pressure. The fraction obtained after evaporation was examined through GC-MS [48]. The equipment used was a Model QP 2010 series from Shimadzu, Tokyo, Japan. The heat was programmed to rise from 50ºC to 300ºC with 2ºC rise per minute. Sample components were ionized in electron impact mode (EI, 70 eV). The temperature of the detector and injector was fixed at 310ºC and 300ºC, respectively, carrier gas Helium was used with a purity of 99.995%. The flow rate was set at 1ml/min with the scanning rate at 3.0 scan/s and mass range of 40 to 1000 m/z. One microliter of source from PHM3 was injected into the GC-MS machine with the help of a Hamilton syringe using split injection (1:40) for total ionic chromatographic analysis. The GC-MS machine was run for 15 min and the analog data was converted to digital data through GC solution software.

### Transesterification of lipids

The standard protocol for the transesterification developed by Christie [49] was used in the current study with certain modifications. Lipids obtained from acid-base extraction of *Pectinodesmus* PHM3, heated in a small beaker placed on the hot plate and any solvent present in it was evaporated at 60 ºC. Methanol (40% v/v) and sulfuric acid (5% v/v) were mixed separately and added to the lipid present in the beaker, 1.5 ml/1500 µl/1.6 g of lipid, 40% /0.7 ml/ 700 µl of methanol and 5%/ 0.075 ml/75µl of sulfuric acid were used. The mixture was placed on the plate for two hours at 60ºC and mixed on a magnetic stirrer at 400 rpm. After 2 h of incubation the mixture was allowed to settle which caused the phases to separate with the lower darker brown phase being biodiesel.

#### Quantification and qualification of alkyl esters through GC-MS

Biodiesel primarily consists of monoalkyl esters with long chain fatty acids [50–52]. To quantify alkyl esters in the biodiesel GC-MS was used as it is highly recommended tool for monitoring organic compounds and is used specifically for the analysis of fatty acids, esters, alcohols, terpenes and aldehydes [53]. Analysis was conducted on a Shimazdu GC-MS QP2020 with SH-Rxi-5Sil MS silica-based capillary column. GC-MS carried an automatic split injector at 250ºC. Solvent used was ethyl acetate. The helium gas carried biofuel at a flow rate of 1.78ml/min. Heat increase was set at 7ºC/min. Initial temperature of 40ºC maintained for 5 min was raised to 300ºC and again maintained for 5 min. All the compounds were identified according to the inbuilt library and analyzed using GC-MS software to determine the total alkyl ester yield [54].

### Lipid processing

The lipid extract from acid base hydrolysis was processed according to the modified phosphatide separation protocols [55, 56]. In this procedure the phosphatides were precipitated by dissolving the lipid extracted in a mixture of super cold acetone and 10% MgCl_2_. 6H_2_O in the ratio of 1:20:0.4, respectively. The mixture was stirred for 15 min and kept at -20^o^C for 2–3 h. After the phosphatide’s precipitation, the suspension was centrifuged and washed; the precipitates were dissolved in chloroform and stored until used the further analysis. FTIR was used to analyze the content of the precipitates.

## Results

### Lipid extraction

**Fig. 2:**
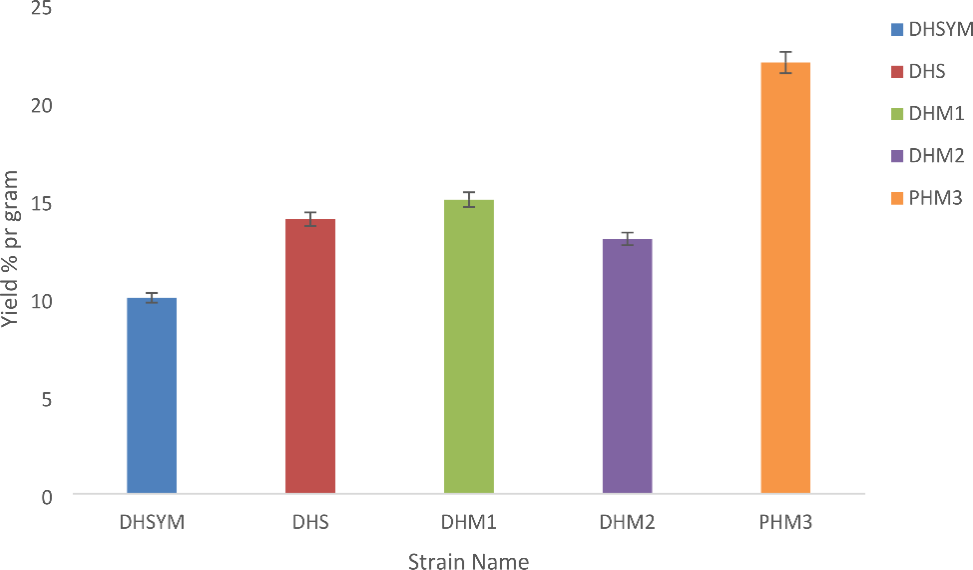
Lipid yield from DHSYM, DHS, DHM1, DHM3 and PHM3 strains. The highest lipid yield is of *Pectinodesmus* (PHM3)

Lipid yield in each strain was measured using Bligh and Dyer method. Among all the algal species, *Pectinodesmus* (PHM3) showed highest average lipid yield of 23% per gram. Due to this reason, all further experiments were conducted with *Pectinodesmus* (PHM3). The comparative analysis of the percentage lipid yield/gram of all five strains is shown in Fig. 2.

**Fig. 3:**
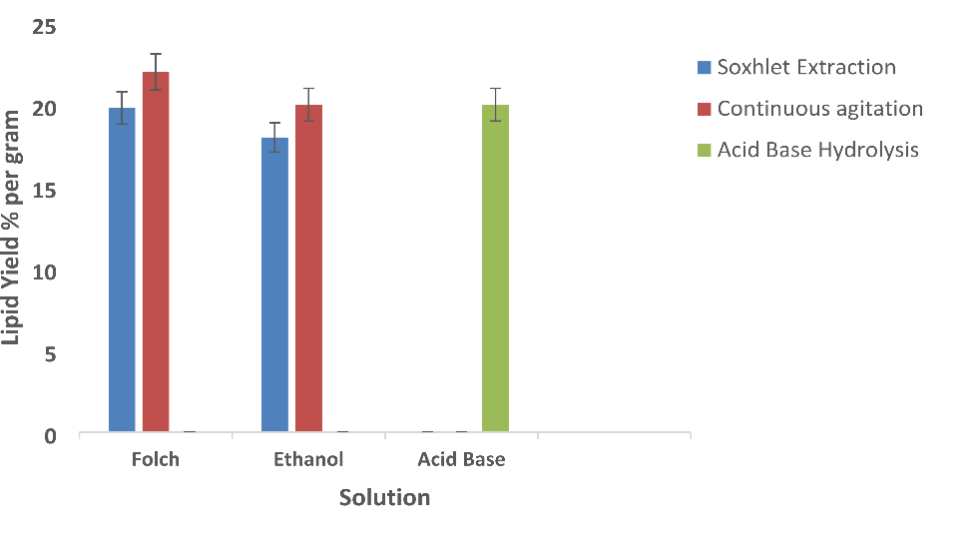
Lipid yield of *Pectinodesmus* PHM3 using Folch extraction method, ethanol with continuous agitation and soxhlet apparatus, and acid base hydrolysis

In this study combination of chemical and mechanistic techniques (Fig. 3) were used to enhance the lipid yield. Highest amount of lipid extracted was through continuous agitation using Folch solution as the highest surface area contact between microalgae biomass and solution occurs through continuous agitation. The second highest lipid levels extracted were from continuous agitation in ethanol solvent and acid base hydrolysis.

### FT-IR study of different lipid extracts

**Fig. 4:**
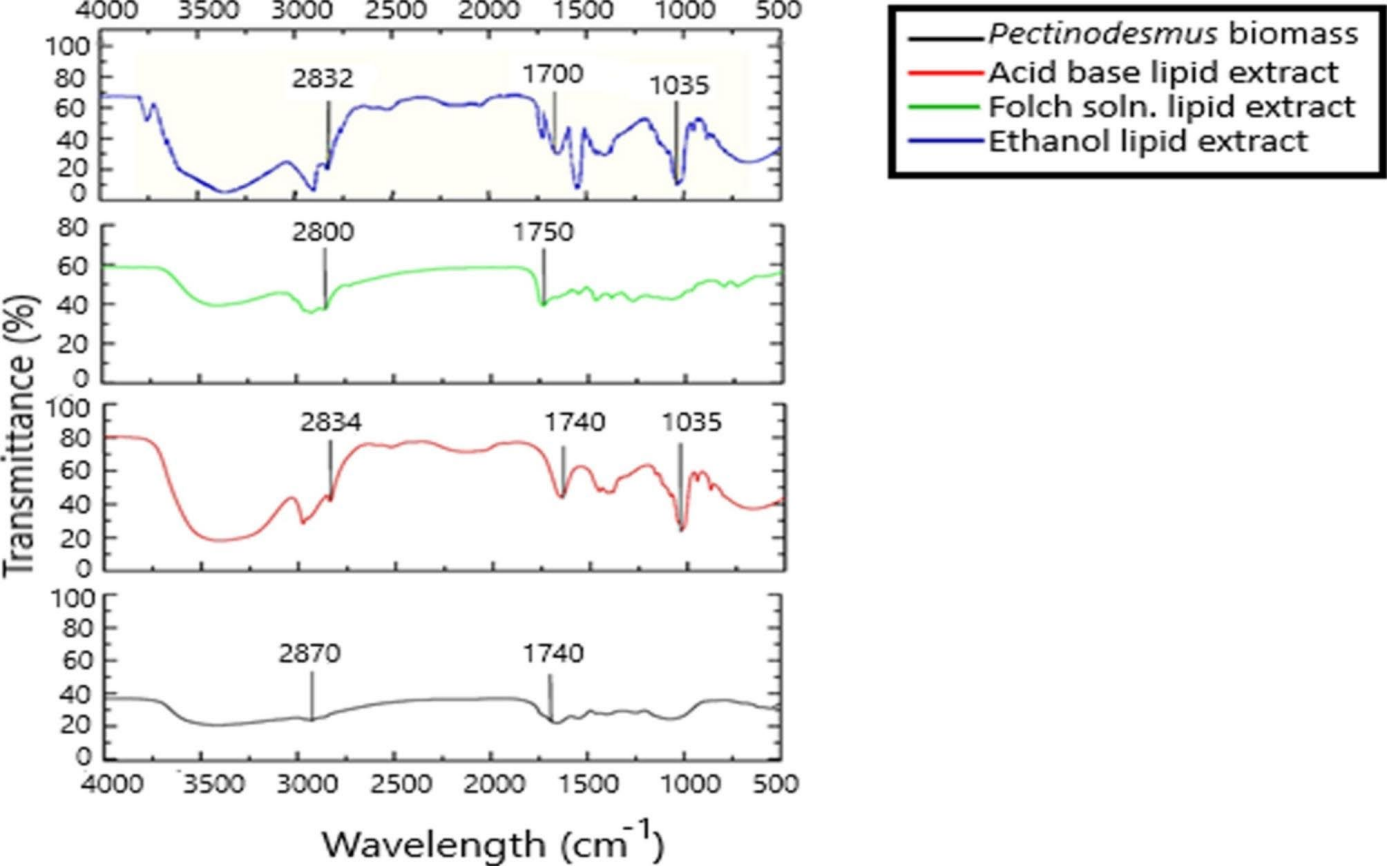
FTIR Spectrum of ethanol, Folch and acid base lipid extracts of *Pectinodesmus* PHM3 in comparison with FTIR to its dry biomass. All peaks between 1700 cm^− 1^ and 2800 cm^− 1^ indicate presence of lipid

Ethanol lipid extract, Folch lipid extract and acid base lipid extract were examined through Fourier transform infrared spectroscopy and compared with the FTIR spectrum of the biomass (Fig. 4).

### Phytochemical analysis of extract

**Fig. 5:**
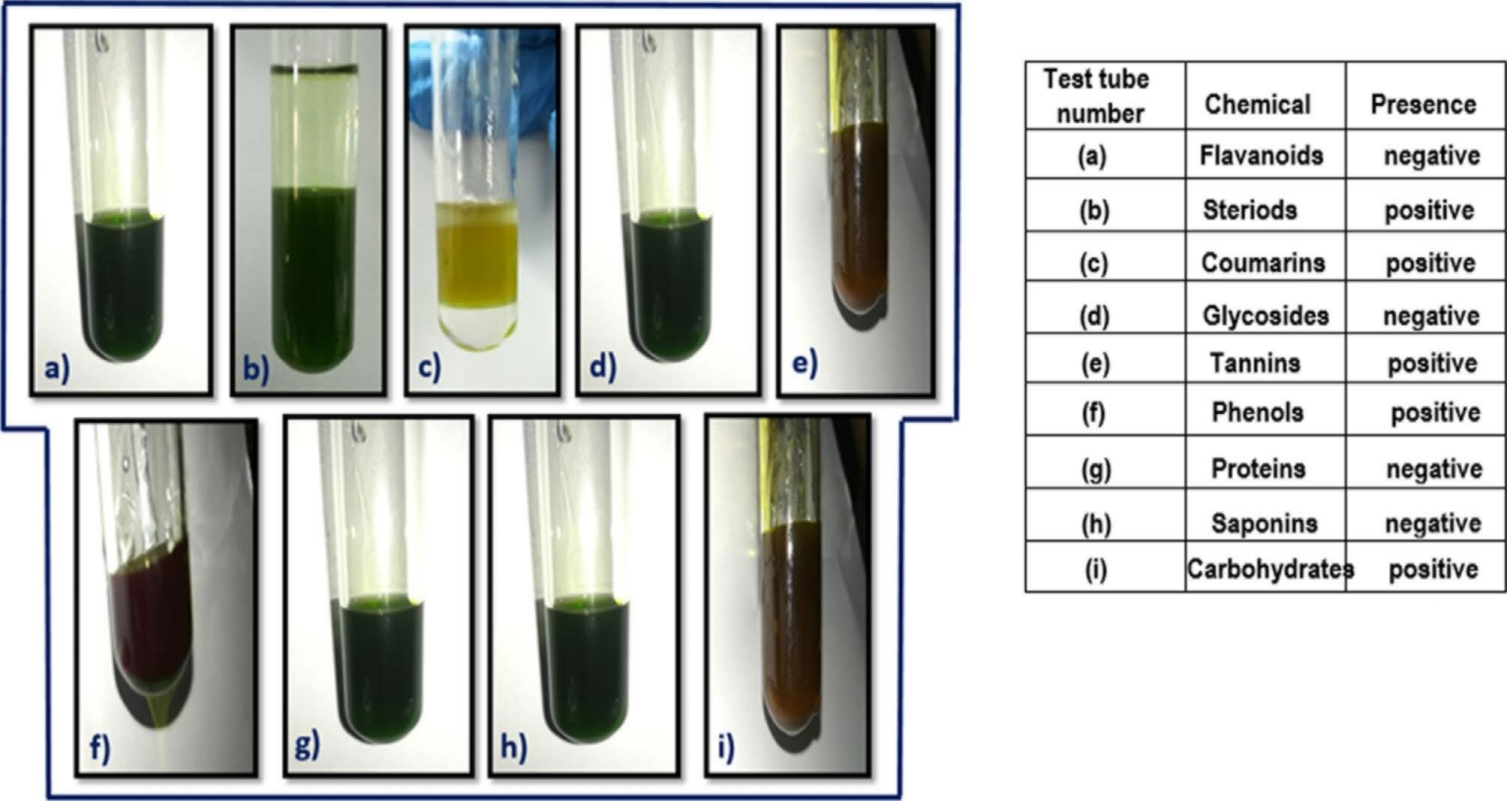
Phytochemical analysis chemical test of *Pectinodesmus* PHM3, presence of these chemicals is represented as **+** and absence is represented as **-** (**a**) Favonoids (-), (**b**) Steroids (+), (**c**) Coumarins (+), (**d**) Glycosides (-), (**e**) Tannins (+), (**f**) Phenols (+), (**g**) Proteins (-), (**h**) Saponins (+), (**i**) Carbohydrates (+)

The phytochemical analysis was conducted in Ethanol which indicated the appearance of a reddish-brown ring over the interface (Fig. 5b), yellow color (Fig. 5c), greenish black color (Fig. 5e), blue green color (Fig. 5f) and brownish tint (Fig. 5i), respectively. Tabular presentation of results are presented in Table 1. Figure 5a confirms the absence of flavonoids while Fig. 5d shows the absence of Glycosides. In addition the test for proteins was shown negative (Fig. 5g) however Saponins presence was confirmed through Phytochemical analysis of *Pectinodesmus* PHM3 (Fig. 5h).

**Table 1:** Major fatty acids and compounds with significant presence in ethanol extract

Peak number	Compound name	Percentage in sample
2 9	Propanoic acid Phosphenous acid	0.16 0.15
35	Glycolic acid	0.7
45	Carboxylic acid	0.29
133	Butenoic acid	0.5
134	Hexenedioic acid	0.29
139	Nonynoic acid	0.15
222	Hexynoic acid	0.15
240	Benzoic acid	0.4
296	Tetradecanoic acid	0.09
324	Hydrastininic acid	0.09
380	Glutaric acid	0.3
501	Fumaric Acid	0.21
513	Diglycolic acid	0.12
514	Octadecanoic acid	0.27
543	Docosanoic acid	0.11
551	Silicic acid	0.2
562	Pentadoic acid	0.15
577	Phtalic acid	0.14
585	Fumaranic acid	0.23
615	Lysergic acid	0.08
622	Succinic acid	0.09
623	Docasanedioic acid	0.12
654	Propylgl	0.1
674	Citamelic acid	0.15
677	Pimelic acid	0.13
680	Heptanoic acid	0.12
692	Hexadecanoic acid	0.13

### GC-MS analysis of PHM3 ethanol extract

**Fig. 6:**
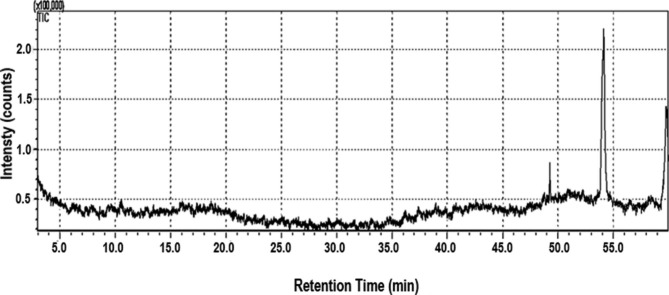
GC-MS chromatogram of ethanol extract. Peak at 54 min indicate the presence of phenol and peak at 59 min confirms the presence of Tris (2,4-di-tert-butylphenyl phosphate

GC-MS analysis of the ethanol extract shown in Fig. 6; Table 1 indicated the presence of a mass of compounds. Some substances were present in high amount in the chromatogram. Phenol was recorded to make total of 8% of sample whereas Tris (2,4-di-tert-butylphenyl) phosphate made 1.4% of the sample as seen by the two high peaks in Fig. 6. The rest of the compounds were in minor amounts and the type and quantity of fatty acids in the extract are given in Table 1.

### Transesterification of lipid

In order to avoid the subsequent saponification as a result of hydrolysis of esters due to water generation from the reaction between hydroxide and alcohol during basic catalyzed reactions, sulfuric acid was used as catalyst for one step transesterification of algal lipids. The use of acid catalysts contributes excessive yields of alkyl esters, but the reaction is not usually fast.

Through the equation applied given in Sect. 2.7 biodiesel yield of *Pectinodesmus* (PHM3) was observed to be 7% per gram dry weight.

#### GC-MS analysis of biodiesel

**Fig. 7:**
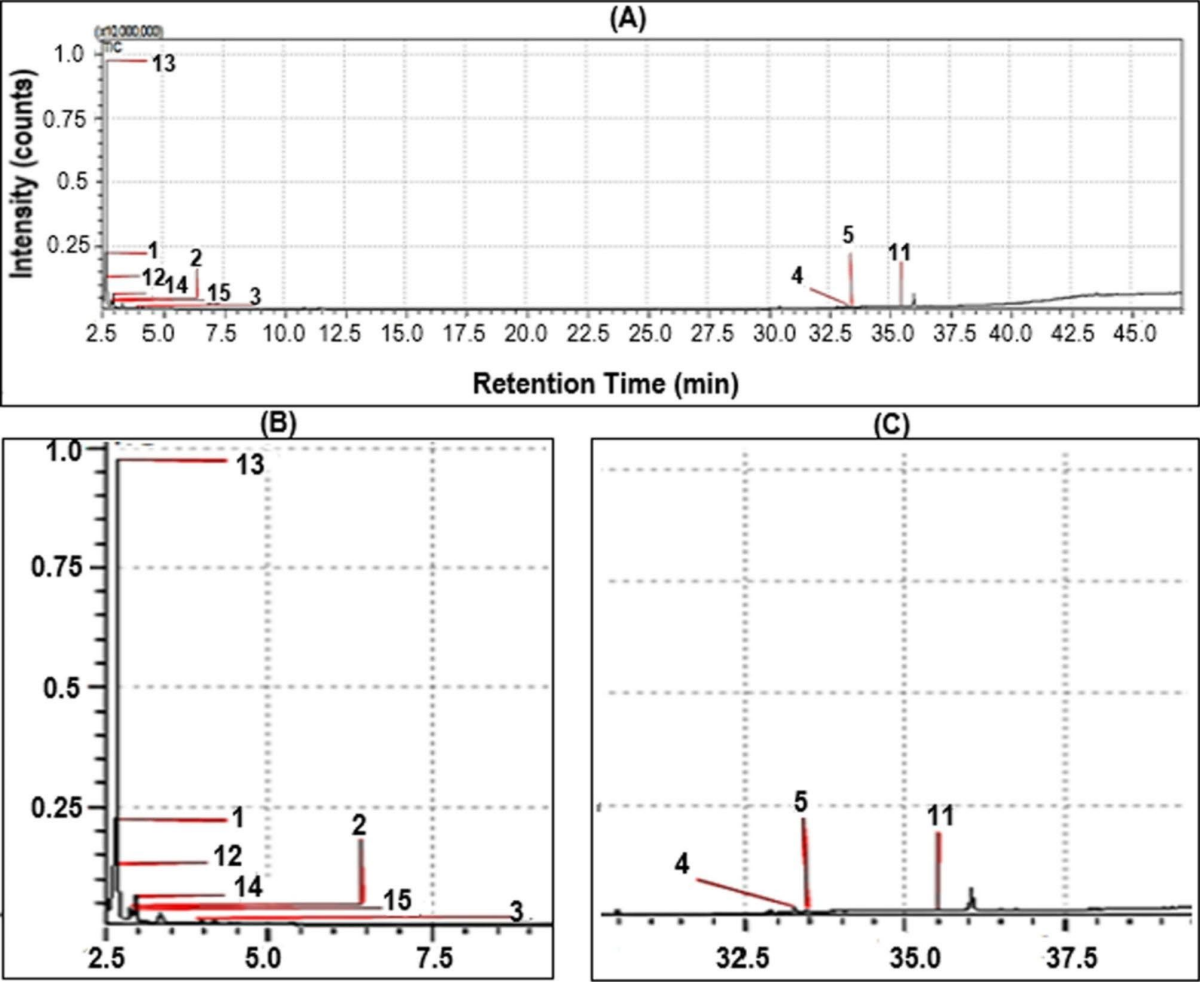
GC-MS analysis. (**A**) GC-MS Chromatogram of biodiesel Trans-esterified from Acid Base lipid extract, (**B**) Visible peaks listed in Table 2, (**C**) Visible peaks listed in Table 2

GC analysis was performed to investigate the chemical composition of biodiesel produced from *Pectinodesmus* PHM3. The peaks in the chromatograms (Fig. 7) of biodiesel samples were analyzed and their respective retention time was used to detect and measure the peaks. Fatty acids details present in the biodiesel produced from Pectinodesmus PHM3 are given in Table 1.

**Table 2:** Major fatty acids present in the biodiesel produced from *Pectinodesmu*s PHM3 with high level of ethers

Sr no	Compound name	Percentage in sample
	**Saturated fatty acids**	
1	Propanoic acid	5.42
2	Acetic acid	0.20
3	Butyric acid	0.24
4	Palmitic acid	1.68
5	Stearic acid	1.3
6	Carbonic acid	0.15
7	Carboxylic acid	0.07
8	Glutaric acid	0.05
	**Unsaturated fatty acids**	
9	Fumaric acid	0.04
	**Others**	
10	Silicic acid	0.32
11	Sulfurous acid	1.56
	**Ethers**	
12	Di propyl ether	4.76
13	Ethyl butyl ether	60
14	Methyl butyl ether	3.30
15	Propyl butyl ether	1.56

Total Saturated Fatty Acids: 20%

Total Unsaturated Fatty acids: 1%

Ethers: 72%

Others:7%

### Lipid processing

**Fig. 8:**
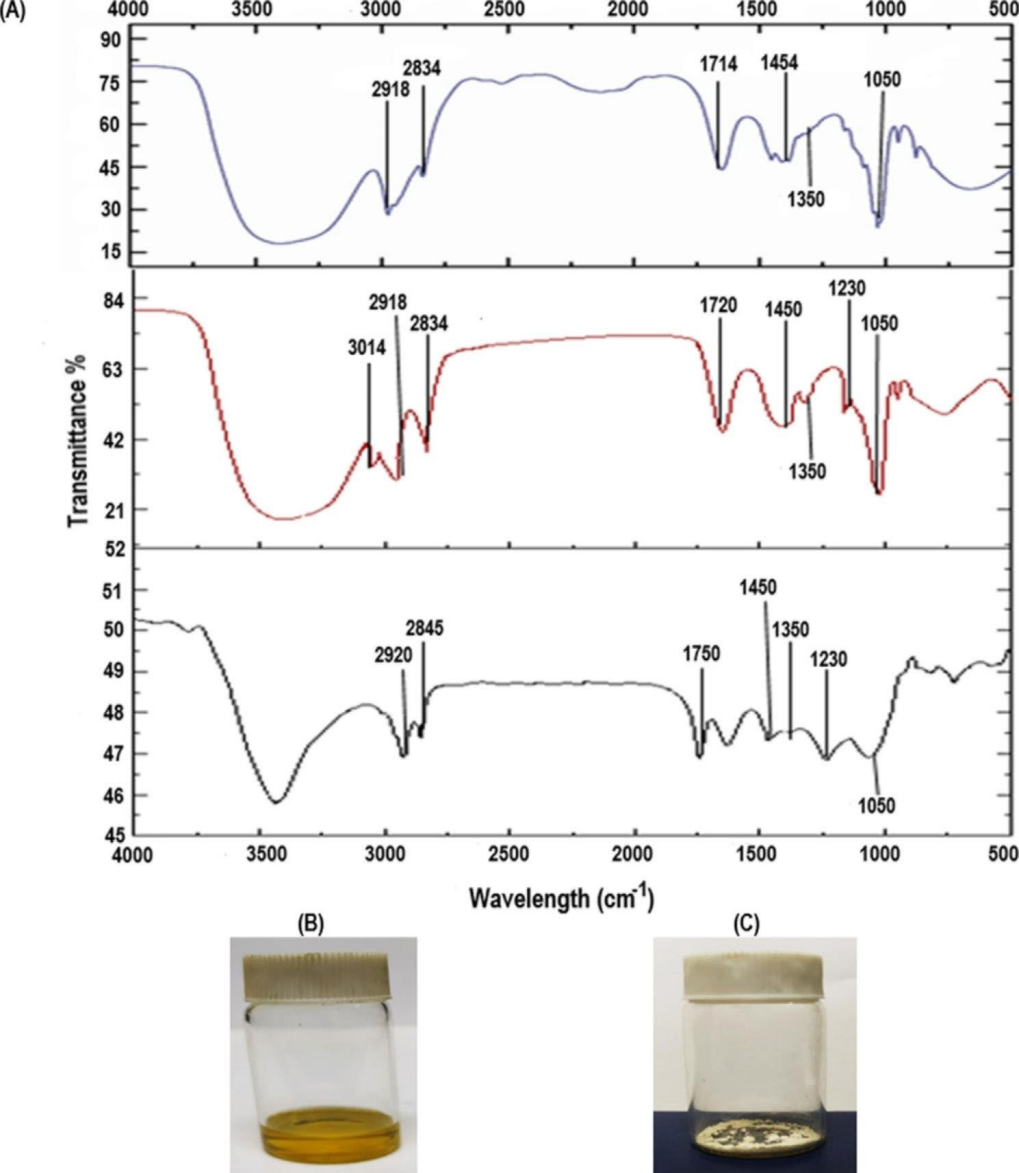
FTIR analysis of lipid extracts. (**A**) FTIR spectra of lipid extracts after acetone processing (**B**) Lipid extract from acid base method (**C**) Lipid extract after processing through acetone precipitation

For lipid processing, FTIR analysis of lipid extracts from ethanol, Folch solution and acid base hydrolysis was performed. As shown in Fig. 8, after proceeding of lipids, there was a major difference in the lipid type, which was closer to phosphatides than before processing (Fig. 3). Figure 8 (b) and (c) showed lipid sample before and after-processing lipid sample. It was observed that oily nature of the lipid was shifted to a more precipitate form which is a common observation when a mixture of lipids is converted to phosphatides.

## Discussion

As already reported microalgae have a diverse biochemical profile. While lipid accumulation is a primary criterion for biodiesel production. In microalgae cellular growth is inversely correlated with lipid production. For a high lipid profile in algae various growth factors require optimization [89–91]. Biofuels from microalgae are the most promising substitute to fossil fuels. The problem arises with evaluating lipid content in all microalgae species. The green microalga *Dictyochloropsis splendida* extract produced the highest lipid and biodiesel yield (12.5 and 8.75% respectively) compared to that of red and brown macroalgae [92].

Among all the algal species, *Pectinodesmus* (PHM3) showed highest average lipid yield of 23% per gram as shown in Fig. 2. Previously, the highest lipid yields recorded are 75–80% (*Schizochytrium* sp. and *Botryococcus braunii* sp.) with the average being 15–20% lipid per gram [14, 15, 57]. The lipid yield of *Pectinodesmus* (PHM3) reported in the current study is in the average range as reported in the previous studies.

In this study, a combination of chemical and mechanistic techniques as shown in Fig. 3 were employed to enhance the lipid yield. Highest amount of lipid extracted was through continuous agitation using Folch solution followed by continuous agitation and acid-base hydrolysis respectively. A previous study reported the total lipid content of 16.7 ± 7.9% dry weight extracted from *Chlorella protothecoides* using Folch solution [58]. Another study has reported an increase in the lipid extraction efficiency of 8.4% in *Tetraselmis* and 5.5% in *Nannochloropsis* using microwave assisted Folch extraction method [59].

FTIR spectra in multiple studies have proved that peaks at a wavelength of 1700–2800 cm^− 1^ correspond to the presence of lipids in the sample [59, 60]. The absorption peaks in the region of 1740 cm^− 1^-1750 cm^− 1^ and 1700 can be correlated to the stretching vibration of carbonyl group of glyceryl ester and carbonyl group of free fatty acids, respectively [61]. Various weaker peaks in the region of 2800 cm^− 1^ can be assigned to the presence of C-H [62]. FT-IR of the biomass shown in Fig. 4 indicates two miniscule peaks at 1740 cm^− 1^ and 2870 cm^− 1^. These are implicative of the presence of carbonyl of glycerol esters and C-H in the biomass. Similarly, peaks observed at 2834 cm^− 1^ and 1740 cm^− 1^ in acid base extract and at 2800 cm^− 1^ and 1740 cm^− 1^ in Folch solution extract can also be correlated to the presence of C-H and carbonyl group of glycerol esters, respectively. Similarly, the peaks at 2832 cm^− 1^ and 1700 cm^− 1^ in the ethanol extract of lipid can be assigned to the presence of C-H and carbonyl group of free fatty acids. A major peak is present at 1035 cm^− 1^ in the acid base extract and ethanol extract is indicative of the presence of phosphatides i.e. polar lipids, as they are known to form peak between 1018 cm^− 1^ and 1050 cm^− 1^ [63]. The Folch extract graph does not show the presence of any polar lipids or peaks in the 1000 cm^− 1^ range because Folch solution removes nonpolar lipids through its methanol solvent [64].

Ethanol is not a purely lipid extraction solvent like chloroform and extracts multiple substances such as proteins, tannins, steroids and phenols, hence the extract would contain a certain amount of other compounds as well [65]. The phytochemical analysis to test presence of steroids and coumarin indicated the appearance of a reddish-brown ring over the interface (Fig. 5b) and appearance of yellow color (Fig. 5c), respectively which confirmed the presence of steroids and coumarin. Similarly, the presence of tannins, phenols and carbohydrates was confirmed through the appearance of greenish black color (Fig. 5e), blue green color (Fig. 5f) and brownish tint (Fig. 5i), respectively. The results also showed the absence of flavonoids, glycosides, proteins and saponin. Such presence of steroids, flavonoids, triterpenes, glycosides, phenols, coumarins and steroids etc., have been reported via phytochemical analysis of microalgae in multiple studies [66–69].

The GC-MS analysis showed high amount of phenol in extracts which indicated that the extract was high in oxidative properties. Such high concentration of phenols in microalgae extracts with anti-oxidative properties have been reported in multiple studies [70–72]. Other major acids present in the extract are glycolic acid, butenoic acid, benzoic acid, glutaric acid, hexenedioic acid, octadecanoic acid. It can be seen from Table 1 majority of the fatty acids like hexanedioic acids, butenoic acid, phosphenous acid etc. present in the ethanol extract are carboxylic acids which are commonly used in the production as antimicrobials in pharmaceutical industry [73]. Similar composition of fatty acids in ethanol extract has also been reported previously [74]. Transesterification results confirmed higher biodiesel yield of *Pectinodesmus* PHM3 compared to an average yield. Previous studies have shown that percentage diesel content in *Jania rubens*, *Galaxaura oblongata*, *Gelidium latifolium*, *Asporagopsis taxiformis* and *Ulva laetuea* was found to be 0.25 ± 0.01%, 2.06 ± 0.02%, 1.3 ± 0.0%, 3.64 ± 0.10% and 3.8 ± 0.12%, respectively [75].

GC-MS analysis of biodiesel obtained from *Pectinodesmus* PHM3 indicated high quantity of unsaturated fatty acids or TAG’s which were converted to biodiesels or ethers i.e. 72% biofuels in the form of dipropyl ethers, ethyl butyl ethers, methyl butyl ether, propyl butyl ether. This shows a high transesterification rate and good biodiesel content considering that average fatty acids methyl esters (FAME) contents of microalgae are between 10 and 20% [76, 77] and the highest FAME contents recorded are around 85% in *Botryococcu*s *braunii* [78]. Moreover, results also showed that total amount of saturated fatty acids is lower than ethers and total saturated fatty acids are negligible i.e. only 1%. The chromatogram created by the machine is available in Fig. 7 and partial list of substances analyzed by gas chromatography are listed in the Table 2. Contents of the biodiesel indicate that *Pectinodesmus* PHM3 has high ether content which can be used in biofuel industry. Previous reports have suggested that alkyl ethers were more beneficial compared to esters and alcohols with respect to all fuel characteristic categories. It was noted that an ether, which comprises of less oxygen, releases less soot per mass. Another examination of fatty alkyl ethers concerning the effect of the length of the alkyl group showed that the increase of carbon number normally augmented all properties studied [79]. Methyl butyl ether and ethyl butyl ether are already being used in the automobile industry [80].

For lipid processing, FTIR analysis of lipid extracts from ethanol, Folch solution and acid base hydrolysis was performed. FTIR spectra of all three extracts were compared in detail to confirm high similitude with lecithin, acid base extract followed by ethanol extract showed most of its peaks matching the peaks of chlorophyll and amino reported by Chang et al. [81] and Wojasinski et al. [82], respectively. The Folch extract showed a high amount of lipid however little none phosphatide prescence. Similarly, FT-IR spectrum of acid base extract showed that there were least amount of protein content and no traces of chlorophyll when compared with FTIR spectra of chlorophyll reported by Chnag et al. [81]. The acid base lipid hydrolysis usually removes chlorophyll and maximizes lipid presence. In case of ethanol lipid extracts, ethanol dissolves multiple types of compounds as according to its alcoholic nature so the number of impurities are higher. In Folch extract, although the solution dissolves the most lipid content, the choloroform in the solution does not dissolve the polar lipids i.e., lipids like phosphatides. The extract after being treated with acetone was again tested with FTIR and there was a shift in the peaks. The phosphatide peak at 1050 cm^− 1^ was more defined and there was significant reduction in noise in the lipid range between 1700 cm^− 1^ and 2800 cm^− 1^. Most of the peaks which showed minor curves before processing showed more defined peaks after processing like the 3014 cm^− 1^, 2918 cm^− 1^, 1450 cm^− 1^, 1350 cm^− 1^ and 1250 cm^− 1^ peak.

## Conclusion

The current study focused on screening five algal strains based on the best yield of lipids. Resultantly, highest amount of lipid was extracted from *Pectinodesmus* PHM3 strain and was found to be 23% per gram. FT-IR spectra of the algal biomass, Folch solution, acid base and ethanol extract showed the presence of glyceryl esters, carboxylic acids and phosphatides. The ethanol extraction of lipids and phytochemical analysis of the ethanol extract indicated the presence of steroids and coumarin, tannins, phenols, and carbohydrates as impurities. The biodiesel yield of *Pectinodesmus* PHM3 was 7% per gram dry weight with the FAME content of the strain to be 72%. Thus, *Pectinodesmus* PHM3 has a biodiesel yield much higher compared to rest of the microalgae strains and exhibits potential for biodiesel production at industrial scale. These experiments were conducted by culturing algae on a small laboratory scale equipment. For biofuel production these lipid extracts need to be further processed. Future endeavors to explore microalgae will help in understand the potential of Pectinodesmus species for biodiesel production. The optimization is however required for large scale production of biodiesel in-order to overcome fuel deficiency.

## Data Availability

The datasets used and/or analysed during the current study are available from the corresponding author on reasonable request.

## Abbreviations

FTIR: Fourier Transmission Infrared Spectroscopy,
GC-MS: Gas Chromatography-mass spectrometry
CO2: Carbon dioxide
FAME: Fatty acids methyl esters
